# Prevalence and Determinants of Male Adolescents’ Smoking in Iran: An Explanation Based on the Theory of Planned Behavior

**DOI:** 10.5812/ircmj.3378

**Published:** 2013-03-05

**Authors:** Mahmood Karimy, Shamsaddin Niknami, Ali Reza Heidarnia, Ibrahim Hajizadeh, Ali Montazeri

**Affiliations:** 1 Department of Health Education, Faculty of Medical Sciences, Tarbiat Modares University, Tehran, IR Iran; 2 Department of Biostatistics, Faculty of Medical Sciences, Tarbiat Modares University, Tehran, IR Iran; 3 Mental Health Research Group, Mother and Child Health Research Centre, Iranian Institute for Health Sciences Research, ACECR, Tehran, IR Iran

**Keywords:** Smoking, Adolescence

## Abstract

**Background:**

Adolescent smoking problem has still remained as a public health concern, but factors that attributing to the initiation of adolescent smoking are not well known in Iran.

**Objectives:**

The aim of this study is to estimate the prevalence of smoking, and its associations among high school male adolescents in Iran, in the context of the theory of planned behavior (TPB).

**Patients and Methods:**

This was a cross-sectional study involving male adolescent students (high school) in the city of Zarandieh, Iran. A multiple-stage sampling protocol was used. The participants completed an anonymous, voluntary, and self-report questionnaire. Prevalence was estimated, and demographic variables, psychological factors, and the theory of planned behavior components were used to indicate factors contributing to adolescents’ cigarette smoking.

**Results:**

In all, 365 students were entered the study. The mean age of respondents was 16.49 ± 1.11 years. The prevalence of current smoking was 15.1%. The result obtained from logistic regression analysis revealed that all theory of planned behavior (TPB) components [knowledge (OR = 0.75; 95% CI: (0.59-0.97), attitude (OR = 0.75; 95% CI: (0.65-0.86), self-efficacy (OR = 0.82; 95% CI: (0.71-0.95), subjective norms (OR = 0.84; 95% CI: (0.72-0.98)] were significant predating factors for adolescents smoking habits. In addition, having parents who smoke (OR = 4.75; 95% CI: (1.38-12.35), smoking friends (OR = 3.76; 95% CI: (1.20-11.76), and smoking siblings (OR = 4.21; 95% CI: (1.17-11.16) were significant contributing factors to adolescents’ cigarette smoking behavior.

**Conclusions:**

The results showed that the prevalence of cigarette smoking in adolescents was high, and the theory of planned behavior (TPB) components were significant predictors of cigarette smoking. It seems that interventions targeting adolescents’ smoking habits might benefit using the TPB model.

## 1. Background

Smoking is still a major health problem among youngsters and adolescents, and between 80% and 90% of adult smokers report having started smoking before reaching the age of 18 (1). Multinational tobacco industry has made the targeting of adolescence for increased tobacco use a priority (2).Youth-specific marketing strategies include sales of single cigarettes or other tobacco products, sponsoring cultural and sporting events with large youth fan bases, advertising near schools, advertising in youth-oriented media, and other similar approaches. These strategies have proven successful as many as 100,000 youth per day begin to use tobacco worldwide, where approximately one-fourth of them are younger than 10 years (2). Studies assessing the behavioral consequences of smoking among the youth have revealed significant links between adolescent smoking and frequent smoking in early adulthood, problems related to or dependency in adulthood, physical health problems, sleep disturbances, academic difficulties, and mental health problems (3). However to explain factors contributing to adolescents smoking habits or to plan programs to prevent smoking among adolescents, several health education models were applied. As such the theory of planned behavior, including peer influences, smoking attitudes and self-efficacy, were commonly used in the theoretically based prevention programs for adolescents (4, 5). The theory of planned behavior is designed to predict and explain human behavior in specific contexts, and in recent years a growing body of research has applied the theory of planned behavior (TPB) to smoking behavior. For smoking, the theory of planned behavior assumes that attitude, self-efficacy, and social norm predict intention to start smoking (4). The findings of the Global Youth Tobacco Survey (GYTS) in Iran in 2008 showed that 5.1% of male students were currently smoking cigarettes (6). According to a recent study in Iran, the prevalence of self-reported cigarette smoking in ages between 17 and 19 years was 12.1% in boys, and 5.3% in girls (7). In the five recent years, rapid changes have occurred in rural areas in Iran including the social, and built environment with the development of population and urbanization, which might have impacted people's lifestyle including smoking behaviors. Whether, to what extent, and in which ways the above factors attribute to the initiation of adolescent smoking are not well known. It is necessary to investigate this important issue to develop more effective smoking prevention programs. In the absence of accurate data on factors associated with smoking among Iranian adolescents, the objective of this study was to identify the determinants of cigarette-smoking among male adolescents in a small town in central part of Iran (Zarandieh).

## 2. Objective

The aim of this study is to estimate the prevalence of smoking, and its associations among high school male adolescents in Iran, in the context of the theory of planned behavior (TPB).

## 3. Patients and Methods 

### 3.1. Design and Data Collection

This cross-sectional descriptive study was performed in Zarandieh city, located in the Markazi Province of Iran, in 2011. The Markazi Province is located in the center of Iran, and comprises 12 cities. The Zarandieh city is located in north of Markazi Province, and has a population of 62000 inhabitants, corresponding to the fifth of the population of the Province. The adolescent population is estimated at 2749 inhabitants. The prevalence of cigarette smoking in the Markazi Province is 15.1%, which this rate is high compared to the mean of cigarette smoking in Iran (8). The study involved 365 students, from high schools and, from the 8th, 9th, and 10th grades, and 15 to 19 years of age. The questionnaire was distributed among the participants, and to ensure the data privacy, school teachers had to leave the classroom during the survey period. Also sufficient time was given to the respondents to fill in the questionnaire. It took around 40-45 minutes to fill in the entire questionnaire.

### 3.2. The Study Questionnaire

A 59-item questionnaire was used to collect the data. The questionnaire was consisted of 3 parts including items on socio-demographic, behavioral constructs, and psychological factors. Here a brief description of the questionnaire is provided:

1. Socio-demographic items: This part was derived from the Global Youth Tobacco Survey (GYTS) developed by the Centers for Diseases Control (CDC, Atlanta, Georgia, USA) (6). The GYTS questionnaire contains 90 multichoice questions, 54 of them are core questions uniformly used in every country. In this study, we used 20 items from the GYTS questionnaire. These included items on age, level of study, class grade, pocket money, parents smoking, friends smoking, parental education, family members’ smoking, and students’ smoking habit, and smoking history. These included the record of the students’ current level of smoking (smoking for a day or more during the past 30 days) , past cigarette smoking experience even for one or 2 puffs (ex-smoker), and nonsmoker (students who never smoked) (6). 

2. Behavioral constructs: This section contained items on knowledge, attitude, subjective norms and self-efficacy. Knowledge toward smoking consisted of 10 items derived from the available literature (7-9). Students responded on a four-point scale ranging from ‘definitely not’ to ‘definitely yes’. The scale was then recoded into a dichotomous variable (0 = maybe/definitely not, and 1 = maybe/definitely yes). The mean of 10 items was calculated to determine the knowledge score. Higher scores indicate higher level of awareness of the harmful effects of smoking. Attitude toward smoking was measured consisted of six items using five-point semantic differential scales, ranging from 0 (strongly agree) to 5 (strongly disagree) taken from the available literature (9, 10). Subjective norms (a person's beliefs about what significant others believe or do) were measured using 6 items recommended by Ajzen, (11) including items on asking whether important others (friends, teachers, siblings, religion, and parents) would approve his smoking habit rated on a 5-point scale ranging from 0 (strongly agree) to 5 (strongly disagree). Self-efficacy was measured using six items derived from the available literature (12, 13) Self-efficacy refers to adolescents’ confidence in their ability to become (or stay) nonsmokers, and their confidence that they could refuse a cigarette when one was offered. The response categories ranged were from ‘very difficult’ to ‘very easy’(3). Psychological items: This part included items on self-esteem, and perceived vulnerability. Self-esteem was measured by using the Rosenberg’s five interval semantic differential scale; (14) rated on a 5-point scale ranging from 0 (strongly agree) to 4 (strongly disagree). Perceived vulnerability, consisted questions derived from the available literature (15). The scale contained two variables of perceived stress (3 items), and perceived depression (4 items). The possible response categories were always, sometimes, rarely, and never. It was rated on a four-point scale ranging from 0 (never) to 3 (always). As for the interpretation of scoring, higher scores indicated a high level of perceived vulnerability. The internal reliability of the questionnaire was examined using the Cronbach’s alpha coefficients, and for all constructs we found satisfactory results (knowledge = 0.85, attitude = 0.94, self-efficacy = 0.87, subjective norms = 0.86, self-esteem = 0.84 and perceived vulnerability = 0.79).

### 3.3. The Study Sample

The study population was included all high school male students, and the required sample for the study was calculated based on an anticipated current smoking prevalence of 9%. The minimum number calculated was 350 (16). To recruit the sample, a multistage (random) probability method was used. 1) The primary sampling units were all high schools (7 high schools). 2) After schools had been recruited, any school quota from the sample was determined based on the ratio of the number of students in each school. 3) Based on the ratio of the number of students in each grade level (1-3) to be determined quota any classroom from sample. 4) The students were randomly selected from these classes based on their identification number. However, the number of students from each school was calculated based on the number of students of each school. Also, from each school, the quota of students from each level (1-3) was chosen. All students attending the school the day of the survey in the selected classes were eligible to participate. Student participation was voluntary and anonymous using self-administered data collection procedures. Data collection was conducted in each school by trained assistants without the presence of the teacher.

### 3.4. Data Analysis

The questionnaires were reviewed and entered a database constructed using the SPSS software, version 16.0. Descriptive variables are expressed as frequency, mean, and overall range (minimum and maximum). The 95% CI was calculated for the precision of prevalence estimates. An independent sample t-test was used to compare the mean scores of knowledge, attitude, subjective norms, self-efficacy, self-esteem, and perceived vulnerability of those who were current smokers, and those who were nonsmoker. To assess the determinant factors of cigarette smoking, multiple logistic regression analysis was applied. Only those independent variables that showed significant associations with smoking (P ≤ 0.05) in uni-variate analysis were included in the multiple logistic regression models. In the logistic regression for determining the dependent variable, Smoking status within 1 month was asked using one question: ‘During the past 30 days (one month), on how many days did you smoke cigarettes?" The responses were dichotomized such that the participants who indicated that they did not smoke or did not smoke in the past 30 days were considered nonsmokers, and coded 0, and those who reported smoking at least one cigarette in the past 30 days were classified as smokers and coded 1.

### 3.5. Ethics

Permission to conduct the study was sought, and obtained from the Ministry of Education, and from the superintendents and school authorities in Markazi Province.

## 4. Results

The mean age of the students (n = 365) was 16.49 ± 1.11 years. The prevalence of smoking experimentation was 44.6 % (163/365). The mean age at smoking experimentation was 13.2 ± 1.9. The most smoking experimentation occurred at 12-13 years in 15.6%, and at 14-15 years in 15.1% of the sample. More than a half of the students (59.4%) had experimented cigarettes before the age of 13. We found that 15.1% of the students were current smokers. Of these, 87.2% believed that smoking is harmful to health (n = 48), 72.3% had tried to quit smoking (n = 40), 89.0% believed they are able to quit smoking (n = 49), and 78.1% intended to quit smoking (n = 43). When the current smokers were asked: Have you ever received advice to help you stop smoking?” 44% of them respond: No, I have never received any advice or help (n = 24). The findings showed that the mean score of knowledge, attitude, self-efficacy, subjective norms, and self-esteem for the nonsmokers was higher than the current smokers, whereas the current smokers perceived a fairly high level of perceived stress and depression (Table 1). The independent sample t-test indicated significant differences between the mean score of all variables of theory of planned behavior (knowledge, attitude, self-efficacy, and subjective norms), and psychological factors (self-esteem, and perceived vulnerability) those who were current smoker, and those who were nonsmoker (P < 0.05). As Table 1 illustrates, the factors that were consistently associated with current smokers in the univariate analysis, included having smoker friends, having smoker parents, and siblings, father’s education, independent room, age, knowledge, attitude, self-efficacy, subjective norms, self-esteem, and perceived vulnerability. The multiple unconditional logistic regression analysis revealed that a number of demographic variables (smoking of friends, parents, and siblings), knowledge, attitude, subjective norms, and self-efficacy were significant factors in predicting cigarette smoking. Having parents who smoke (OR = 4.75; 95% CI: (1.38-12.35), smoking siblings (OR = 4.21; 95% CI: (1.17-11.16), smoking friends (OR = 3.76; 95% CI: (1.20-11.76) were the most important predictors of smoking (Table 1).

**Table 1.** Results of theUni-variate and Multiple Logistic Regression Analysis

Demographics	Current smokers (n = 55)	Non-smokers (n = 310)	OR (95% CI)^a^	P	OR (95% CI) ^b^	P
**Age, y, Mean ± SD**	16.92 ± 1.11	16.42 ± 1.09	1.51 (1.15-1.97)	< 0.002	1.24 (.74-2.09)	0.40
**Smoking friends, No. (%)**						
No	19 (3.83)	201 (54.24)	1.0 (Ref.)			
Yes	36 (11.23)	109 (30.68)	3.49 (1.91-6.38)	< 0.001	3.76 (1.20-11.76)	0.02
**Smoking parents, No. (%)**						
No	22 (6.02)	246 (67.39)	1.0 (Ref.)			
Yes	33 (9.04)	64 (17.53)	2.56 (1.39-4.69)	< 0.002	4.75 (1.38-12.35)	0.01
**Smoking siblings, No. (%)**						
No	43 (10.95)	283 (77.53)	1.0 (Ref.)			
Yes	15 (4.10)	27 (7.39)	3.93 (1.92-8.01)	< 0.001	4.21 (1.17-11.16)	0.02
**Behavioral constructs, Mean ± SD**						
Knowledge,	20.05 ± 2.00	21.73 ± 2.57	0.74 (0.65-0.85)	< 0.001	0.75 (0.59-0.97)	0.02
Attitude	8.98 ± 5.31	16.61 ± 3.69	0.68 (0.62-0.74)	< 0.001	0.75 (0.65-0.86)	0.001
Self-efficacy	9.58 ± 4.29	17.25 ± 3.32	0.66 (0.59-0.72)	< 0.001	0.82 (0.71-0.95)	0.009
Subjective norms	15.63 ± 3.11	20.55 ± 3.24	0.78 (0.72-0.85)	< 0.001	0.84 (0.72-0.98)	0.02
**Psychological factors, Mean ± SD**						
Self-esteem	23.72 ± 7.35	30.06 ± 5.47	0.84 (0.80-0.89)	< 0.001	0.99 (0.91-1.08)	0.98
Perceived stress	5.54 ± 2.22	3.47 ± 2.03	1.70 (1.43-2.02)	< 0.001	1.13 (0.83-1.53)	0.42
**Perceived depression**	6.89 ± 2.28	3.90 ± 2.22	1.65 (1.43-1.90)	< 0.001	1.24 (0.97-1.58)	0.07

^a^Obtained from univariate analysis

^b^Adjusted OR obtained from multiple logistic regression analysis

## 5. Discussion

The 44.6% prevalence of smoking experimentation found among the adolescents in this study can be considered high when compared to what reported in the studies conducted in other Iranian districts (7-9). However, it is difficult to directly compare this percentage with those in other studies that have assessed the prevalence of smoking among Iranian adolescents due to between-study differences in the age, geographic location, and definition of ‘experiment smoking’ of the participants. However, this may also be due to high prevalence (38.6%) of cigarette smoking by family members in this survey, because, family members who smoke can facilitate the smoking habit in adolescents, due to the availability of cigarettes in the home. This finding might also be associated with a trend toward an increase in smoking among Zarandieh adolescents. Unfortunately, in the recent years, multinational tobacco industry has made the targeting of youth for increased tobacco use a priority (2). In Islamic Republic of Iran it is estimated that the population under 15 years old is about 26 million, and this group would be an ideal market for tobacco industry (9). In this study, the mean age at smoking experimentation was 13.2 years. The mean age of high school students who had experimented tobacco in this study is in agreement with the result reported for the students from 35 European countries with the ages ranging from 13 to 15 years; (17) initiation in all places seems to be below 20 years; (18, 19) therefore, it is pertinent that these age groups be targeted to prevent their first puffs, as after that age, the likelihood of starting is very low. Having smoking friend(1) has been considered as the most important factor influencing the habit of smoking in adolescents in the Western world; (20) but in this study, the impact of parents is more important for adolescents smoking behavior. However, Chassin; (21) suggested that the roles of parents and older siblings have more importance for smoking initiation than those of peers. The most straightforward interpretation is that parents who smoke serve as models for the behavior of their children (22). Also, parents who smoke may facilitate their children’s smoking simply by giving them easier access to cigarettes or allowing smoking in home (23). Consistent with other studies; (22, 24) the present study indicated that knowledge variable was a significant factor in predicting cigarette smoking, and that the nonsmokers scored higher on the knowledge variable compared to the current smokers. In contrast, a study by Islam in the Egypt showed that knowledge of the short-term negative consequences of smoking was protective against susceptibility to future smoking among females only (25). Therefore, increasing the knowledge of adolescents through health education programs on smoking could lead to a significant reduction in cigarette smoking among adolescents. Previous researches on adolescent smoking behavior have indicated more positive attitude toward smoking, and that smokers tended to be related with an increased likelihood of smoking (26, 27). Similarly, our study indicated that attitude variable was a significant factor in predicting cigarette use. In general, adolescent smokers have less knowledge about the negative consequences of smoking than their nonsmoking counterparts, discount the addictive property of tobacco, and negate the risks of experimental smoking (5). The findings revealed that subjective norms of the samples could significantly predict their cigarette smoking. Similar findings have been reported in other studies. For instance, Fujimoto investigated Social network influences on adolescent substance use, and found that subjective norms could predict smoking behaviors in American adolescents (28). This result is inconsistent with the study of Nehl, which revealed that subjective norms could not predict African American College Students intention to smoke (29). The present study also indicated that self-efficacy variable was a significant factor in predicting cigarette use. In the smoking literature, low self-efficacy has been related to smoking initiation, and smoking rate as well as greater difficulty in quitting and /or higher rates of relapse among adolescents (12). Some studies conducted in the United States have found that low refusal self-efficacy reduces the ability to say “no” to an offer of a cigarette, which is positively associated with adolescent cigarette smoking (1, 13). The result of this study further indicated that self-esteem variable was not a significant factor in predicting cigarette use, but the mean score of self-esteem for nonsmokers was higher than current smokers. The study of Lazuras in Greek adolescents showed that intention to smoke was stronger among the adolescents with low self-esteem, suggesting that self-esteem may act as a vulnerability factor in the process of smoking initiation (30). The study of Li in Nanjing, China, showed that the low self-esteem was positively associated with current smoking among adolescents.(31). In fact, they may regard smoking as a means of coping with the stress, anxiety, and depression associated with lack of self-confidence. In the present study, there were significant differences between the mean score of perceived stress/depression in the current smokers, and those who were nonsmokers. Also logistic regression analysis showed that with increasing perceived stress/depression level, they have more chances to smoke. Previous studies provided evidence of association between smoking with depression and stress.(32, 33). This may be due to smokers misunderstanding that smoking can help relieve their depression and stress. A study by Weiss on California students identified that smokers were likely to report more depressive symptoms than nonsmokers (34). Several studies have failed to find an association between depression, and subsequent smoking behavior, and have instead found smoking to predict onset of depression (35). It has also been suggested that depression increases adolescents’ vulnerability to pressure from peers to initiate smoking (35). The present study had several limitations: Firstly, it relies on self-completion of the questionnaires, so the accuracy of reporting in this study is not known. Although adolescents were assured of their anonymity, still some students might have been fearful of entrapment, because of the cultural burden of tobacco smoking among adolescents in Iran, and therefore, may have underreported their cigarette smoking. Second, the study participants were recruited from schools. Interpretation of the results to the general adolescent population in Zarandieh must be made with caution as school going adolescents may not represent the overall adolescent population. In conclusion, smoking prevalence in high school adolescents living in Zarandieh is high and our findings provided support for the utility of the predictor variables of the theory of planned behavior (TPB). Knowledge, attitude, subjective norms, and self-efficacy components were all significantly associated with cigarette smoking. The findings also indicated that parental, friends’ and siblings habits are important predictors of adolescents’ cigarette use. Thus, in planning and implementation of educational tobacco control programs; there is the need to the target habits of parents, friends, and siblings as well as the theory of planned behavior (TPB) component as important influencing factors.
